# Long-term safety and impact of immune recovery in heavily treatment-experienced adults receiving fostemsavir for up to 5 years in the phase 3 BRIGHTE study

**DOI:** 10.3389/fimmu.2024.1394644

**Published:** 2024-05-28

**Authors:** Josep M. Llibre, Judith A. Aberg, Sharon Walmsley, Juan Velez, Carlos Zala, Brenda Crabtree Ramírez, Bronagh Shepherd, Rimi Shah, Andrew Clark, Allan R. Tenorio, Amy Pierce, Fangfang Du, Bo Li, Marcia Wang, Shiven Chabria, Michael Warwick-Sanders

**Affiliations:** ^1^ Department of Infectious Diseases, Hospital Universitari Germans Trias i Pujol, Barcelona, Spain; ^2^ Division of Infectious Diseases, Icahn School of Medicine at Mount Sinai, New York, NY, United States; ^3^ University Health Network, Toronto, ON, Canada; ^4^ Medicina Interna – Infectología, Fundación Valle del Lili, Cali, Valle del Cauca, Colombia; ^5^ Department of Microbiology, University of Buenos Aires, School of Medicine, Buenos Aires, Argentina; ^6^ Departamento de Infectología, Instituto Nacional de Ciencias Médicas y Nutrición, Salvador Zubirán, Mexico City, Mexico; ^7^ GSK, Brentford, United Kingdom; ^8^ ViiV Healthcare, Brentford, United Kingdom; ^9^ ViiV Healthcare, Branford, CT, United States; ^10^ ViiV Healthcare, Durham, NC, United States; ^11^ GSK, Collegeville, PA, United States

**Keywords:** ARV, clinical trials, drug resistance, intervention, treatment

## Abstract

**Introduction:**

Fostemsavir is a gp120-directed attachment inhibitor approved for heavily treatment-experienced (HTE) adults with multidrug-resistant HIV-1. We provide detailed week 240 safety results from the BRIGHTE study and evaluate the impact of immune recovery on safety outcomes.

**Methods:**

The phase 3 BRIGHTE trial is ongoing; data for this analysis were collected from the first participant’s first visit (February 23, 2015) through the last participant’s last visit for week 240 (March 22, 2021). Safety endpoints were assessed in participants who received fostemsavir + optimized background therapy. In participants with baseline CD4+ T-cell count <200 cells/mm^3^, exposure-adjusted adverse event (AE) rates were assessed among subgroups with or without CD4+ T-cell count ≥200 cells/mm^3^ at any time during 48-week analysis periods through week 192.

**Results:**

Through a median of 258 weeks (range, 0.14–319) of treatment, discontinuations due to AEs occurred in 30/371 (8%) participants. Serious AEs were reported in 177/371 (48%) participants, including 16 drug-related events in 13 (4%) participants. Thirty-five (9%) deaths occurred, primarily related to AIDS or acute infections. COVID-19–related events occurred in 25 (7%) participants; all resolved without sequelae. Among participants with baseline CD4+ T-cell count <200 cells/mm^3^, 122/162 (75%) achieved CD4+ T-cell count ≥200 cells/mm^3^ at week 192. Exposure-adjusted AE rates were markedly lower among participants achieving CD4+ T-cell count ≥200 cells/mm^3^ at any time vs those sustaining <200 cells/mm^3^. No new AIDS-defining events were reported after week 48 in participants with CD4+ T-cell count ≥200 cells/mm^3^.

**Conclusions:**

Cumulative safety findings through the BRIGHTE 240-week interim analysis are consistent with other trials in HTE participants with advanced HIV-1 and comorbid disease. Reduced rates of AIDS-defining events and AEs were observed in participants with immunologic recovery on fostemsavir-based treatment.

**Clinical trial number:**

NCT02362503, https://clinicaltrials.gov/study/NCT02362503.

## 1 Introduction

Heavily treatment-experienced (HTE) individuals living with HIV-1 have limited treatment options due to resistance and intolerance to multiple prior antiretrovirals (1, 2). For these individuals, the approvals of 3 first-in-class antiretrovirals (the gp120-directed attachment inhibitor fostemsavir (3), the CD4-directed post-attachment inhibitor ibalizumab (4), and the capsid inhibitor lenacapavir (5)) have introduced new treatment options; however, creating viable antiretroviral regimens can be complicated by comorbidities, psychosocial factors, drug-drug interactions, and non-adherence (2, 6–9). An antiretroviral regimen with a favorable safety and tolerability profile is essential for HTE individuals who may have experienced intolerance and/or toxicity issues and who may require concomitant therapies for comorbidities (2, 7, 9).

Compromised immunologic function is not unusual in HTE persons and contributes to an increased risk of adverse clinical outcomes (2, 9). Low CD4+ T-cell count (<200 cells/mm^3^) is associated with increased morbidity and mortality, from both non-specific and specific causes, including COVID-19, tuberculosis, and AIDS-related illnesses (10–14). Low CD4+/CD8+ ratio (<0.3) correlates with higher risk of non–AIDS-defining events and mortality (15–17). Restoration of immunologic function, measured by increases in absolute CD4+ T-cell count to >200 cells/mm^3^ and CD4+/CD8+ ratio to >0.45, is an important treatment goal for all people living with HIV, particularly those with low CD4+ T-cell count (16–20).

In the phase 3 BRIGHTE trial, HTE adults treated with fostemsavir + optimized background therapy (OBT) experienced durable virologic responses and clinically meaningful improvements in CD4+ T-cell count and CD4+/CD8+ ratio through week 240 (21). BRIGHTE included participants with advanced HIV-1 disease: 75% had baseline CD4+ T-cell count <200 cells/mm^3^ (30% had baseline CD4+ T-cell count <20 cells/mm^3^; mean, 138 cells/mm^3^; median, 80 cells/mm^3^), and mean baseline CD4+/CD8+ ratio was 0.18 (8).

CD4+ T-cell responses in BRIGHTE were greater than expected based on prior studies in similar populations (22–24). Mean increase in CD4+ T-cell count from baseline to week 240 was 296 cells/mm^3^ in the randomized cohort (RC) and 240 cells/mm^3^ in the non-randomized cohort (NRC), and mean CD4+/CD8+ ratio increased from 0.20 at baseline to 0.60 at week 240 in the RC and from 0.12 to 0.32 in the NRC (21). Among RC participants with baseline CD4+ T-cell count <50 cells/mm^3^, 74% (34/46) had ≥200 cells/mm^3^ at week 240, representing a potential decreased risk of most AIDS-defining illnesses and a transition from opportunistic infection prophylaxis to no prophylaxis (21, 25).

Here we provide a detailed description of week 240 safety results from BRIGHTE. To better understand the potential impact of immune recovery on safety outcomes in BRIGHTE, we determined exposure-adjusted AE frequency over time by baseline CD4+ T-cell count and among those achieving CD4+ T-cell count ≥200 cells/mm^3^ any time on study vs those sustaining <200 cells/mm^3^ during 48-week analysis periods through week 192.

## 2 Materials and methods

### 2.1 Study design and participants

BRIGHTE (ClinicalTrials.gov, NCT02362503) is a phase 3 trial that included HTE adults (aged ≥18 years) with HIV-1 with virologic failure (screening HIV-1 RNA ≥400 copies/mL) and ≤2 fully active and available antiretrovirals remaining (8, 21, 26). Participants with 1 to 2 fully active antiretrovirals were randomly assigned (3:1) to receive oral fostemsavir 600 mg twice daily or placebo + current failing regimen (RC) for 8 days followed by open-label fostemsavir + OBT for all participants. Participants with no fully active and available antiretrovirals received open-label fostemsavir + OBT starting on day 1 (NRC). In the NRC, the OBT could include investigational antiretrovirals (eg, ibalizumab). BRIGHTE remains ongoing wherever participants are unable to access fostemsavir by other means. For this analysis, the first participant’s first visit occurred on February 23, 2015, and the last participant’s week 240 visit occurred on March 22, 2021; the week 240 data cutoff was June 24, 2021.

BRIGHTE was performed in accordance with the Declaration of Helsinki. Study protocols, amendments, and other required documents were reviewed and approved by the Western Institutional Review Board (Puyallup, WA). All participants provided written informed consent before study initiation.

### 2.2 Safety assessments

Cumulative safety data were collected through the week 240 data cutoff for the safety population (all participants who received ≥1 dose of fostemsavir). Safety assessments included monitoring of AEs, clinical laboratory tests, vital signs, electrocardiograms (ECGs), and physical examinations. Electrocardiograms were used to monitor QTc intervals, including thresholds for stopping study drug (see **Supplementary Methods 1**). Assessments were performed on day 1 and weeks 4, 8, 12, 16, 24, 36, 48, and every 48 weeks thereafter and/or early termination (including week 240).

Adverse events were coded using the Medical Dictionary for Regulatory Activities (MedDRA; version 19.1). Severity of AEs and laboratory abnormalities was graded using the Division of AIDS Table for Grading the Severity of Adult and Pediatric Adverse Events (version 2.0, November 2014) (27). Relationship to study drug was determined by the treating investigator.

Adverse events of special interest (AESIs), selected on the basis of emerging non-clinical/clinical safety data for fostemsavir, disease and/or population events, and/or regulatory requirements, included QTc prolongation/ventricular tachyarrhythmias and immune reconstitution inflammatory syndrome (IRIS).

At the start of the COVID-19 pandemic (December 2019), all ongoing BRIGHTE participants had received ≥192 weeks of fostemsavir + OBT. Investigators used World Health Organization guidelines for COVID-19 diagnosis and reported exposure risk, testing results, and symptom presence (28).

### 2.3 Analyses of adverse events by baseline and on-treatment CD4+ T-cell count

In participants with baseline CD4+ T-cell count <200 cells/mm^3^ and an available CD4+ T-cell count at week 192, a pilot analysis of crude incidence rates of AEs was conducted among subgroups based on week 192 CD4+ T-cell count (<200 vs ≥200 cells/mm^3^). This pilot analysis was limited by a small sample size and a single time point. To include participants who discontinued before week 192 and additional time points for CD4+ T-cell count measurements, another analysis was conducted. This second analysis evaluated exposure-adjusted AE incidence rates calculated using participant-years of fostemsavir exposure for subgroups based on CD4+ T-cell count during four 48-week analysis periods (0 to <48, ≥48 to <96, ≥96 to <144, and ≥144 to <192 weeks) in participants with baseline CD4+ T-cell count <200 cells/mm^3^. Week 192 was used as the data cutoff for these analyses because subsequent results were impacted by study completion and the COVID-19 pandemic (**Table 1**). For each 48-week analysis period, participants were assigned to 1 of 2 subgroups: (1) CD4+ T-cell count remained <200 cells/mm^3^ for all measures throughout the analysis period or (2) CD4+ T-cell count was ≥200 cells/mm^3^ at least once during the analysis period.

**Table 1:** Participant Disposition Through the Week 240 Data Cutoff (June 24, 2021; Safety Population).

Participants, n (%)	Randomized cohort (N=272)	Non-randomized cohort (N=99)	Total (N=371)
At week 240 data cutoff			
Ongoing	133 (49)	23 (23)	156 (42)
Completed^†^	55 (20)	25 (25)	80 (22)
Withdrawn	84 (31)	51 (52)	135 (36)
Primary reason for withdrawal^‡^			
Lack of efficacy	17 (6)	10 (10)	27 (7)
Adverse event	8 (3)	5 (5)	13 (4)
Withdrawal by participant	10 (4)	2 (2)	12 (3)
Death^§^	11 (4)	17 (17)	28 (8)
Lost to follow-up	12 (4)	3 (3)	15 (4)
Non-adherence with study drug	15 (6)	6 (6)	21 (6)
Pregnancy	2 (<1)	0	2 (<1)
No longer meets study criteria	7 (3)	6 (6)	13 (4)
Other^¶^	2 (<1)	2 (2)	4 (1)
At week 192 visit window			
Ongoing	200 (74)	51 (52)	251 (68)
Visit impacted by COVID-19^#^	9 (5)	2 (4)	11 (4)
Completed	0	0	0
Withdrawn	72 (26)	48 (48)	120 (32)
At week 240 visit window			
Ongoing	188 (69)	44 (44)	232 (63)
Visit impacted by COVID-19^#^	44 (23)	15 (34)	59 (25)
Completed	5 (2)	7 (7)	12 (3)
Withdrawn	79 (29)	48 (48)	127 (34)

^†^80 participants completed the study by the time of the week 240 database lock, though only 12 did so before their week 240 observations. ^‡^Each participant may have only 1 primary reason. ^§^A total of 35 participants died. Death was recorded as the reason for withdrawal in 28/35 cases. ^¶^Other reasons for discontinuation were investigator decision, HIV resistance, investigator discretion due to rapid progression of the participant’s malignancy, and participant developed transportation obstacles preventing ongoing participation. ^#^Visit missed or at least 1 assessment missed. Percentage based on number of participants ongoing at the time of the visit window.

Assessed AE categories were all-cause AEs, Centers for Disease Control and Prevention (CDC) class C AIDS-defining events, and infections and infestations. Infections and infestations included preferred terms of special interest (PTSIs) from the MedDRA infections and infestations system organ class (SOC), which were selected to exclude terms associated with non-specific infections and infections that were non-serious and/or typically not related to immunosuppression (see **Supplementary Methods 2**). Exposure-adjusted incidence rates for each analysis period were based on number of reported AEs with a start date in the period and number of participant-years of fostemsavir exposure within the period.

## 3 Results

### 3.1 Participants

Of 371 participants enrolled and treated in BRIGHTE, 232 (63%) were ongoing at their week 240 study visit window (RC, n=188; NRC, n=44). Of these, 59/232 (25%) had their week 240 visit impacted by the COVID-19 pandemic, including 13 (6%) who missed the visit altogether and 46 (20%) who had some assessments missed. At the week 240 data cutoff (June 24, 2021), 156/371 (42%) participants were ongoing, 133/272 (49%) in the RC and 23/99 (23%) in the NRC (**Table 1**).

Baseline characteristics for BRIGHTE participants were consistent with advanced HIV-1 disease, particularly in the NRC (**Supplementary Table 1**). In the RC and NRC, baseline CD4+ T-cell count was <200 cells/mm^3^ for 199/272 (73%) and 79/99 (80%) participants, respectively, and <20 cells/mm^3^ for 72/272 (26%) and 40/99 (40%) participants, respectively. Furthermore, the majority of participants had >10 years of previous antiretroviral treatment, with 92/272 (34%) and 58/99 (59%) participants in the RC and NRC, respectively, having >20 years of treatment.

### 3.2 Analysis of adverse events through week 192 by baseline and on-treatment CD4+ T-cell count

#### 3.2.1 Pilot assessment

At week 192, 162/278 (58%) participants with baseline CD4+ T-cell count <200 cells/mm^3^ had an on-treatment CD4+ T-cell count at week 192 and 122/162 (75%) had CD4+ T-cell count ≥200 cells/mm^3^. Overall, the frequency of serious AEs (SAEs), any grade 3/4 AEs, and AIDS-defining events was lower among participants with CD4+ T-cell count ≥200 cells/mm^3^ at week 192 vs those with <200 cells/mm^3^ (**Table 2**). This was particularly apparent in the RC, while in the NRC, no differences were seen for SAEs or grade 3/4 AEs. No difference between subgroups was seen in either cohort for AEs from the infections and infestations SOC, possibly due to the impact of common non-serious infections (eg, upper respiratory tract infections). A subsequent analysis used only PTSIs, as described in the Methods.

**Table 2:** Summary of AEs Through Week 192 by CD4+ T-Cell Count at Week 192 for Participants With Baseline CD4+ T-Cell Count <200 Cells/mm^3†^.

Participants, n (%)	CD4+ T-cell count <200 cells/mm^3^ at week 192^‡^			CD4+ T-cell count ≥200 cells/mm^3^ at week 192^‡^		
	Randomized cohort (N=21)	Non-randomized cohort (N=19)	Total (N=40)	Randomized cohort (N=105)	Non-randomized cohort (N=17)	Total (N=122)
Any AE	21 (100)	19 (100)	40 (100)	103 (98)	17 (100)	120 (98)
Drug-related AEs	8 (38)	6 (32)	14 (35)	45 (43)	10 (59)	55 (45)
Any grade 3–4 AE	14 (67)	10 (53)	24 (60)	39 (37)	10 (59)	49 (40)
Any SAE	16 (76)	10 (53)	26 (65)	45 (43)	11 (65)	56 (46)
Drug-related SAEs	0	1 (5)	1 (3)	0	1 (6)	1 (<1)
All discontinuations	2 (10)	3 (16)	5 (13)	6 (6)	0	6 (5)
AEs leading to discontinuation	0	0	0	2 (2)	0	2 (2)
AEs in infections and infestations SOC	19 (90)	18 (95)	37 (93)	89 (85)	17 (100)	106 (87)
AIDS-defining events (CDC class C events)	3 (14)	3 (16)	6 (15)	6 (6)	1 (6)	7 (6)
Deaths	1 (5)	1 (5)	2 (5)	1 (<1)	0	1 (<1)

AE, adverse event; CDC, Centers for Disease Control and Prevention; SAE, serious AE; SOC, system organ class.

^†^Table only includes participants who had a week 192 CD4+ T-cell count as well as a baseline CD4+ T-cell count <200 cells/mm^3^; for this reason, numbers are low in most groups due to discontinuation. ^‡^Week 192 was used as the data cutoff for these analyses because subsequent results were impacted by study completion and the COVID-19 pandemic.

#### 3.2.2 Exposure-adjusted adverse event assessment

**Figure 1:**
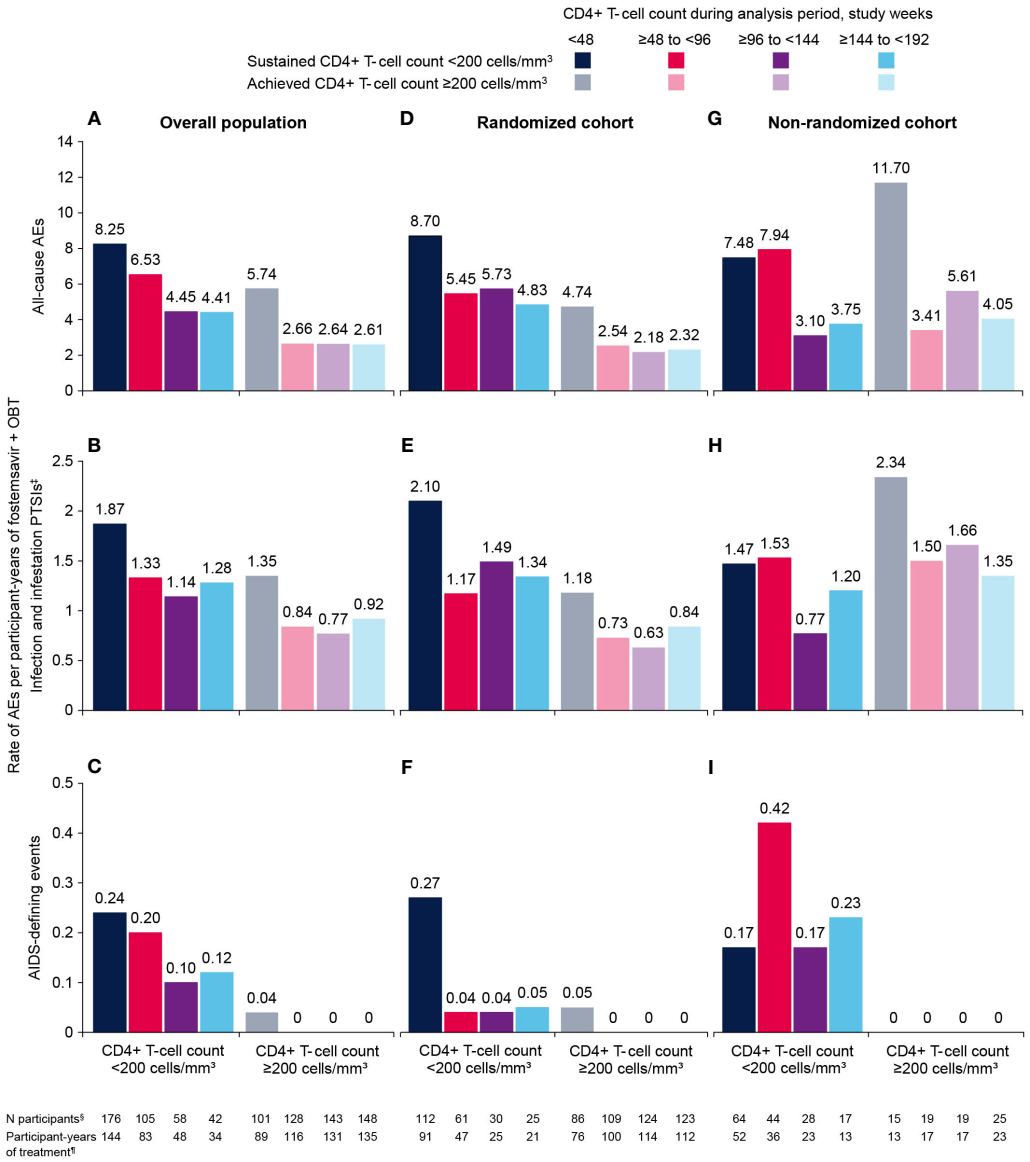
Exposure-adjusted rates of **(A, D, G)** all-cause AEs, **(B, E, H)** infection and infestation PTSIs, and **(C, F, I)** AIDS-defining events through week 192^†^ by CD4+ T-cell count during the analysis period for participants with baseline CD4+ T-cell count <200 cells/mm^3^. In participants randomized to placebo, only data from start of open-label fostemsavir are included. For each panel, AE rates are based on the number of reported AEs for the specified category (y-axis) with a start date within the specified analysis period (legend). AE, adverse event; OBT, optimized background therapy; PTSI, preferred term of special interest. ^†^Week 192 was used as the data cutoff for these analyses because subsequent results were impacted by study completion and the COVID-19 pandemic. ^‡^Infectious disorders and ectoparasitic infestations. Infections and infestations included PTSIs from the Medical Dictionary for Regulatory Activities infections and infestations system organ class, which were selected to exclude terms associated with non-specific infections and infections that were non-serious and/or typically not related to immunosuppression. ^§^Number of participants who received fostemsavir, had ≥1 CD4+ T-cell count during the specified analysis period, and had CD4+ T-cell count <200 cells/mm^3^ for all measures throughout the specified analysis period (<200 subgroup) or had at least 1 measure of ≥200 cells/mm^3^ at any time during the specified analysis period (≥200 subgroup). ^¶^Total number of participant-years of treatment with fostemsavir for subgroup participants over the specified analysis period.

Overall, exposure-adjusted rate of all-cause AEs was markedly lower among participants with CD4+ T-cell count ≥200 cells/mm^3^ at any time compared with those with sustained CD4+ T-cell count <200 cells/mm^3^ during the same analysis period (**Figure 1**). Similar patterns were seen for infection and infestation PTSIs and AIDS-defining events. In participants with any on-treatment CD4+ T-cell count ≥200 cells/mm^3^ at least once within each 48-week analysis period, no new AIDS-defining events were reported after week 48. For all types of AEs, the highest exposure-adjusted rates occurred in the first 48 weeks of the study, with lower frequencies observed in subsequent analysis periods. Results in the RC were similar to those for the overall analysis.

In the NRC, numbers of participants with on-treatment CD4+ T-cell count ≥200 cells/mm^3^ were very low in all analysis periods compared with the RC. There were no clear differences in exposure-adjusted rates of all-cause AEs or infection and infestation PTSIs between subgroups; however, AIDS-defining events in the NRC occurred only in participants with sustained CD4+ T-cell count <200 cells/mm^3^ through week 192. Furthermore, frequency of all-cause AEs and infection and infestation PTSIs decreased after week 96.

### 3.3 Cumulative safety through week 240

At the week 240 cutoff, median duration of fostemsavir exposure for the safety population was 258 weeks (range, 1 day to 319 weeks). Total participant-years of exposure to fostemsavir was 1428.28 (RC, 1101.33; NRC, 326.95). Cumulative safety through week 240 in BRIGHTE has previously been reported (21) and is presented in **Table 3**. Across both cohorts, 357/371 (96%) participants reported ≥1 AE. Adverse events led to discontinuation in 30 (8%) participants.

**Table 3:** Cumulative Safety Through Week 240.

Participants, n (%)	Randomized cohort (N=272)	Non-randomized cohort (N=99)	Total (N=371)
Overview of AEs			
Any AE	259 (95)	98 (99)	357 (96)
Any grade 2–4 AE	242 (89)	94 (95)	336 (91)
Drug-related grade 2–4 AEs	65 (24)	23 (23)	88 (24)
Any grade 3–4 AE	110 (40)	60 (61)	170 (46)
Any SAE^†^	122 (45)	55 (56)	177 (48)
Drug-related SAEs^‡^	10 (4)	3 (3)	13 (4)
AEs leading to discontinuation^§^	17 (6)	13 (13)	30 (8)
CDC class C events	25 (9)	19 (19)	44 (12)
Most common CDC class C events^¶^			
Candidiasis esophageal (definitive diagnosis)	4 (1)	4 (4)	8 (2)
Candidiasis esophageal (presumptive diagnosis)	3 (1)	4 (4)	7 (2)
Pneumocystis pneumonia (clinically diagnosed)	5 (2)	2 (2)	7 (2)
HIV wasting syndrome	0	6 (6)	6 (2)
Recurrent pneumonia	3 (1)	1 (1)	4 (1)
CMV retinitis	0	3 (3)	3 (<1)
Histoplasmosis (disseminated)	3 (1)	0	3 (<1)
Immunoblastic sarcoma	0	3 (3)	3 (<1)
Pneumocystis pneumonia (histologically proven)	1 (<1)	2 (2)	3 (<1)
Progressive multifocal leukoencephalopathy (proven)	3 (1)	0	3 (<1)
Cryptococcosis extrapulmonary	0	2 (2)	2 (<1)
HIV dementia	2 (<1)	0	2 (<1)
Deaths	15 (6)	20 (20)	35 (9)
Reported cause of death, n (% of deaths)			
AIDS-related	4 (27)	8 (40)	12 (34)
Acute infections	8 (53)	4 (20)	12 (34)
Non-AIDS malignancies	3 (20)	3 (15)	6 (17)
Other causes	0	5 (25)	5 (14)
COVID-19 diagnoses^#^	19 (7)	6 (6)	25 (7)
Confirmed with positive PCR test	15 (6)	3 (3)	18 (5)
COVID-19 reported as an SAE	7 (3)	0	7 (2)
Any ventricular tachyarrhythmia-related AESIs^‖^	13 (5)	1 (1)	14 (4)
Electrocardiogram QT prolonged	5 (2)	1 (1)	6 (2)^††^
Syncope	6 (2)	0	6 (2)^‡‡^
Loss of consciousness	2 (<1)	0	2 (<1)
Ventricular tachycardia	1 (<1)	0	1 (<1)
Grade 3 or 4 laboratory changes^§§,¶¶^			
Estimated creatinine clearance^##^	128 (48)	38 (39)	166 (45)
Creatinine	93 (35)	24 (24)	117 (32)
Direct bilirubin	28 (10)	14 (14)	42 (11)
Triglycerides^‖‖^	16 (7)	8 (11)	24 (8)
ALT	18 (7)	2 (2)	20 (5)
AST	13 (5)	4 (4)	17 (5)
Total cholesterol^‖‖^	15 (7)	1 (1)	16 (5)
Total bilirubin	9 (3)	6 (6)	15 (4)
Creatine kinase	12 (4)	3 (3)	15 (4)
Urate	10 (4)	5 (5)	15 (4)

AE, adverse event; AESI, AE of special interest; ALT, alanine aminotransferase; AST, aspartate aminotransferase; CDC, Centers for Disease Control and Prevention; CMV, cytomegalovirus; IRIS, immune reconstitution inflammatory syndrome; MedDRA, Medical Dictionary for Regulatory Activities; PCR, polymerase chain reaction; SAE, serious AE.

^†^SAEs occurring in ≥2% of participants were pneumonia (n=25), cellulitis (n=10), acute myocardial infarction (n=8), acute kidney injury (n=8, all with identified reversible causes not related to study drug), COVID-19 (n=7), sepsis (n=6), and coronary artery disease (n=6). ^‡^Drug-related SAEs (16 events in 13 participants) included IRIS (n=3); nephrolithiasis (n=2); and n=1 each of acute kidney injury, hyperglycemia, hyperkalemia, loss of consciousness, myocarditis, hepatocellular cytolysis, rhabdomyolysis, fetal growth restriction, disorientation, and rash through the week 96 data cutoff and supraventricular tachycardia (n=1) after the week 96 data cutoff. ^§^The most common AEs leading to discontinuation were related to infections (n=12); 4 participants discontinued because of an AE after the week 96 cutoff (1 each for pneumonia, cytomegaloviral pneumonia, polyneuropathy, and rash). ^¶^CDC class C events occurring in 2 or more participants in a cohort. ^#^Diagnosis based on World Health Organization definition. ^‖^AEs identified under the standardized MedDRA query Torsades de Pointes/QT prolongation (broad), which includes all preferred terms in ICH E14 with the exception of seizure. ^††^Protocol-specified QTc prolongation stopping criteria were met by 4/6. ^‡‡^No reports of syncope were considered related to study treatment, and 5/6 were non-serious; 1 serious event followed an acute myocardial infarction and a cerebrovascular incident and resolved during the study. ^§§^Graded according to Division of AIDS Table for Grading the Severity of Adult and Pediatric Adverse Events, version 2.0 (Nov 2014). ^¶¶^Unless otherwise specified, N=268 for the randomized cohort, N=99 for the non-randomized cohort, and N=367 for the overall population. ^##^N=98 for the non-randomized cohort and N=366 for the overall population. ^‖‖^N=221 for the randomized cohort, N=73 for the non-randomized cohort, and N=294 for the overall population.

Serious AEs and deaths were more frequent in the NRC than the RC. These were most commonly reported from the infections and infestations SOC. A total of 16 drug-related SAEs were reported in 13 participants, with 15 occurring before week 96. The only drug-related SAEs reported more than once were IRIS (3 cases) and nephrolithiasis (2 cases), and only 3 participants discontinued because of a drug-related SAE (**Supplementary Table 2**).

There were 35 deaths (9%; RC, n=15; NRC, n=20), including 6 (3 per cohort) that occurred after week 96 (**Table 3**; **Supplementary Table 3**). All but 1 of the participants who died were treated with fostemsavir. Deaths were primarily related to AIDS (n=12) or acute infections (n=12). One death was considered by the investigator to be related to study drug (IRIS related to recurrent atypical mycobacterial infection). Only 2/35 participants who died had a baseline CD4+ T-cell count >200 cells/mm^3^, while most (26/35 [74%]) had <50 cells/mm^3^ (median baseline CD4+ T-cell count, 11 cells/mm^3^). The last recorded CD4+ T-cell count before death was <200 cells/mm^3^ in 32/35 (91%) participants who died.

During the study, 25 participants had 28 COVID-19–related events. Four participants had suspected COVID-19, but no confirmatory polymerase chain reaction (PCR) test result was reported, 3 had negative COVID-19 PCR test results reported, and 18 had PCR-confirmed COVID-19 (RC, n=15; NRC, n=3). All cases resolved without reported sequelae, and there were no reports of post–COVID-19 syndrome (although this was not specifically queried). Seven participants (all in the RC) were hospitalized, and COVID-19 was reported as an SAE (**Supplementary Table 4**). For these participants, the most recent CD4+ T-cell counts before COVID-19 diagnosis ranged from 164 to 1641 cells/mm^3^, and 5/7 had HIV-1 RNA <40 copies/mL. Median event duration was 19 days (range, 15–43). Treatment for COVID-19 often included prophylactic anticoagulants and supplemental oxygen; no changes were made to any antiretroviral regimen.

#### 3.3.1 QTc prolongation

Eleven (3%) participants were discontinued from the study for meeting protocol-specified ECG QTc prolongation stopping criteria (7 before the week 96 cutoff; **Supplementary Figure 1**). Of these 11 discontinuations, 1 participant was receiving concomitant amiodarone for atrial fibrillation, 2 had QTc prolongation at baseline, and 7 had at least 1 confounding ECG abnormality present at baseline (eg, sinus bradycardia, left or right bundle branch block, left axis deviation, and left anterior fascicular block). In 4/11 cases, the investigator reported the ECG QT prolongation as an AE (all non-serious). None experienced a corresponding symptomatic cardiovascular event or had any evidence of ventricular tachyarrhythmia, and most (9/11 [82%]) were able to continue fostemsavir treatment after study discontinuation. Two additional cases of ECG QT prolongation were reported as AEs but did not result in discontinuation (**Table 3**).

#### 3.3.2 Immune reconstitution inflammatory syndrome

IRIS was reported in 8 (2%) participants; all cases occurred within 16 weeks of treatment initiation (**Supplementary Table 5**). Five events occurred in participants with baseline CD4+ T-cell count <20 cells/mm^3^. Three events were classified as SAEs, including 1 associated with recurrent atypical mycobacterial infection that was fatal; no other events resulted in discontinuation of study treatment.

#### 3.3.3 Laboratory abnormalities

The most common grade 3/4 laboratory abnormalities reported through week 240 were increased serum creatinine, decreased estimated creatinine clearance, and increased direct bilirubin. Mean increase in serum creatinine and/or decrease in estimated creatinine clearance was apparent from week 4, after which there were continued small changes through week 240. Participants with emergent grade 3/4 changes in serum creatinine (n=117) and/or estimated creatinine clearance (n=166) typically had other risk factors for renal decline including comorbidities, low baseline CD4+ T-cell count, and low baseline body weight. Concomitant medications associated with increased serum creatinine included sulfamethoxazole/trimethoprim (used by 188/371, 51%) and azithromycin (used by 166/371, 45%). Further, 305/371 (82%) participants included dolutegravir in their initial OBT, many twice daily (242/371, 65%); increased serum creatinine is a known effect of dolutegravir, which inhibits renal transport of creatinine via organic cation transporter 2 (OCT2) (29). All participants with emergent grade 3/4 changes in creatinine and/or creatinine clearance were able to continue fostemsavir.

There were no emergent grade 4 increases in direct bilirubin. Grade 3 direct bilirubin elevation was reported in 42 participants. Ten of these participants experienced a hepatobiliary AE/SAE during the study: 3 with hepatitis B virus reactivation; 1 with hepatitis C virus infection and concurrent increases in alanine aminotransferase (ALT) and aspartate aminotransferase (AST); and 1 each with acute hepatitis A infection, cholangiocarcinoma, cirrhosis, cholelithiasis with hepatosteatosis, liver abscesses due to extrapulmonary tuberculosis, and Hodgkin’s disease with liver metastases. In the remaining cases, most increases in direct bilirubin were transient, without clinical signs or manifestations of liver disease, and resolved without discontinuation of fostemsavir. Three participants discontinued the study because of hepatobiliary AEs (hepatic failure [2 cases], hepatic cytolysis [1 case], and hepatorenal syndrome [1 case]).

#### 3.3.4 Pregnancies

Through the week 240 data cutoff, 6 participants became pregnant (although the protocol required the use of contraception). Per protocol and after a benefit–risk assessment by the investigator, participants were permitted to continue fostemsavir. Three pregnancies led to normal births of healthy infants with no complications after exposure to fostemsavir either in all 3 trimesters (2 cases) or the first trimester (1 case). Two pregnancies, with exposure to fostemsavir in all 3 trimesters, had complications (1 fetal growth restriction and 1 premature birth) but led to otherwise normal births of infants with no congenital abnormalities. One pregnancy ended in an elective abortion.

## 4 Discussion

Analysis of long-term data over a median follow-up of 258 weeks in BRIGHTE shows that fostemsavir was well tolerated with a safety profile that was consistent with previous results (8, 26). Infections and HIV-1 disease progression accounted for the majority of grade 3/4 AEs, SAEs, and deaths, likely reflecting the severity of immune compromise in the study population.

The remarkable CD4+ T-cell recovery seen at earlier time points was sustained through longer-term follow-up, with 75% of participants with baseline CD4+ T-cell count <200 cells/mm^3^ achieving ≥200 cells/mm^3^ at week 192. Exposure-adjusted rates of all-cause AEs, infection and infestation PTSIs, and AIDS-defining events were lower among participants with CD4+ T-cell count ≥200 cells/mm^3^ any time during the analysis period compared with those with sustained CD4+ T-cell count <200 cells/mm^3^. These differences were less apparent in the NRC, which was smaller than the RC population (n=99 vs n=272) and also had a higher proportion of participants with more advanced HIV-1 disease. Notably, in the NRC, there were no emergent AIDS-defining events reported among participants with any on-treatment CD4+ T-cell count ≥200 cells/mm^3^. The improved immune function also likely explains the absence of serious sequelae or deaths among BRIGHTE participants who had COVID-19 infections.

The immunologic responses seen during treatment with fostemsavir-based regimens in BRIGHTE may be related to its unique mechanism of action. Temsavir, the active component of fostemsavir, binds directly to both membrane-associated and soluble gp120, preventing the entry of HIV-1 into host cells, and also possibly inhibiting antibody-dependent cytotoxicity of bystander CD4+ T-cells and activation of other downstream inflammatory pathways that may contribute to CD4+ T-cell death (30). Immunologic non-responders are an important population of people living with HIV who experience insufficient CD4+ T-cell count recovery despite persistent virologic suppression and have a higher risk of AIDS and non–AIDS-defining events and malignancies, as well as an increased risk of mortality (31–34). Attempts to improve CD4+ T-cell count in this population through antiretroviral treatment intensification with maraviroc and raltegravir have been unsuccessful (35–38). The potential role of fostemsavir in immunologic recovery is the subject of ongoing and future studies.

In BRIGHTE, there were 5 pregnancies with exposure to fostemsavir that were carried to term and resulted in live births of infants with no congenital abnormalities. Although there were very few cases and more data are needed, there are HTE individuals of childbearing potential who may benefit from fostemsavir.

Protocol-defined stopping criteria for QTc prolongation were met by 11 participants; 7 had ECG abnormalities at baseline, none had corresponding symptomatic cardiovascular events or evidence of ventricular tachyarrhythmia, and 9 continued fostemsavir treatment after discontinuing from the study. In healthy volunteers, a fostemsavir dose of 1200 mg once daily did not produce clinically important QTc interval prolongation (defined as the upper bound of the 2-sided 90% CI of ddQTcF exceeding 10 ms); a supratherapeutic fostemsavir dose of 2400 mg twice daily resulted in a mean increase in QTcF interval of 11.2 ms (upper bound of 90% CI, 13.3 ms) (39).

Eight cases of IRIS occurred, all before week 96, 3 of which were serious. HIV-associated IRIS is a known complication in individuals who start antiretroviral therapy with advanced immunosuppression (as is the case with our study population) and is associated with considerable morbidity and mortality (18, 19).

Through week 240, there were no new clinically relevant laboratory toxicities. Although 32% of treated participants experienced an emergent grade 3/4 serum creatinine elevation, results were confounded by identifiable risk factors for reduced renal function such as advanced HIV-1 disease, concomitant medications, and comorbidities. Many participants received concomitant medications known to influence creatinine (eg, sulfamethoxazole/trimethoprim), and the initial OBT for >80% of participants included dolutegravir (29). A causal relationship between fostemsavir and elevation in serum creatinine has not been established (3). There were no emergent grade 4 increases in direct bilirubin, and grade 3/4 increases in total bilirubin were infrequent. Most cases of grade 3 direct bilirubin elevation were minor, transient, asymptomatic, and resolved without discontinuation of fostemsavir. All 4 participants with ALT ≥3× ULN and total bilirubin ≥2× ULN had overlapping clinical events commonly associated with hepatobiliary inflammation.

Two unavoidable limitations of BRIGHTE, given the treatment needs of the target population, are the lack of a control arm beyond the initial short-term blinded period and variability of the individualized OBT. The complex clinical characteristics of the BRIGHTE population, with advanced HIV-related disease and multiple comorbidities with concomitant medications, further complicate interpretation of the results.

Cumulative safety findings through the BRIGHTE 240-week interim analysis show that fostemsavir has a favorable long-term safety and tolerability profile, attributes that are important for HTE individuals, who have few remaining treatment options, may have advanced disease and multiple comorbidities, and use complex treatment regimens. The reduced rates of AIDS-defining events and AEs in participants who achieved CD4+ T-cell count recovery highlight the importance of achieving immune recovery with a salvage regimen in the HTE population, as was seen in BRIGHTE. Additionally, fostemsavir has a drug-drug interaction profile that allows co-administration with most drugs prescribed for HIV-1 and associated comorbidities without dose adjustment (3, 40) and favorable tolerability in individuals with renal or hepatic impairment (41). These attributes, coupled with the robust week 240 virologic and immunologic response rates (21), support fostemsavir as an important treatment option for HTE individuals with multidrug-resistant HIV-1.

## Data availability statement

The data sets presented in this article are not readily available due to privacy reasons. Anonymized individual participant data and study documents can be requested for further research from www.clinicalstudydatarequest.com.

## Ethics statement

The study involving humans was approved by Western Institutional Review Board (Puyallup, WA). The study was conducted in accordance with the local legislation and institutional requirements. The participants provided their written informed consent to participate in this study.

## Author contributions

JL: Writing – review & editing, Formal analysis, Investigation. JA: Writing – review & editing, Formal analysis, Investigation. SW: Writing – review & editing, Formal analysis, Investigation. JV: Investigation, Formal analysis, Writing – review & editing. CZ: Formal analysis, Writing – review & editing, Investigation. BCR: Formal analysis, Investigation, Writing – review & editing. BS: Formal analysis, Writing – review & editing, Conceptualization, Methodology. RS: Conceptualization, Writing – original draft, Formal analysis. AC: Conceptualization, Writing – review & editing. AT: Formal analysis, Writing – review & editing. AP: Formal analysis, Writing – review & editing. FD: Formal analysis, Writing – review & editing. BL: Formal analysis, Writing – review & editing. MW: Formal analysis, Writing – review & editing. SC: Formal analysis, Writing – review & editing. MW-S: Formal analysis, Writing – review & editing.
