# Supplementary material for: Long-term safety and impact of immune recovery in heavily treatment-experienced adults receiving fostemsavir for up to 5 years in the phase 3 BRIGHTE study
Source: Front Immunol. 2024 May 28;15:1394644. doi: 10.3389/fimmu.2024.1394644 (PMC11165140; doi:10.3389/fimmu.2024.1394644)
Supplement: Supplementary file 1 [file DataSheet_1.docx]

Supplementary Material

# Supplemental Methods: Withdrawal Criteria

Premature discontinuation was required for participants who met any 1 of the following conditions during the study:

- Participant request to stop study treatment
- Any clinical adverse event, laboratory abnormality (including positive HIV RNA), or intercurrent illness that, in the opinion of the investigator, indicated that continued participation in the study was not in the best interest of the participant
- Level of viremia that, in the opinion of the investigator, indicated that continued participation in the study was not in the best interest of the participant (eg, accounting for limited available future options, risks of clinical deterioration)
- Termination of the study by ViiV Healthcare
- Loss of ability to freely provide consent through imprisonment or involuntary incarceration for treatment of either a psychiatric or physical (eg, infectious disease) illness
- Unblinding a participant for any reason (emergency or non-emergency)
- Repeat non-adherence by the participant with the requirements of the protocol or treatment
- Participant required discontinuation of fostemsavir for reasons of intolerability, safety, or efficacy
- Development of potential drug-induced liver injury
- Withdrawal of informed consent (participant decision to withdraw for any reason)
- Pregnancy, unless continuing was considered by the investigator to be in the best interest of the participant
- Confirmed QT value >500 ms
- Confirmed QTcF value >470 ms for female participants and >450 ms for male participants, or confirmed increase in QTcF value >60 ms over baseline, regardless of sex, unless the QTcF prolongation was believed secondary to a reversible cause (ie, a non-essential concomitant medication) that could be readily acted upon and allowed for the timely return of the QTcF interval to below the discontinuation threshold (>470 ms for female participants, >450 ms for male participants, and >60 ms increase over baseline). Regardless of the suspected underlying cause of QTcF prolongation, whenever there was evidence of QTcF prolongation (>470 ms for female participants, >450 ms for male participants, or increase in QTcF >60 ms over baseline) by computer-generated or manual assessment of an electrocardiogram (ECG) collected in a study participant, the ECG was repeated in triplicate (a total of 3 ECGs collected over a brief time period; ≤10 minutes). When multiple ECGs were collected in triplicate, the mean QTcF interval of those tracings was used to help determine the participant’s status in the study. All collected ECGs were submitted for standardized assessment by the central reading group. All instances of QTcF prolongation with the potential for per-protocol discontinuation of a participant from the study required direct communication between the investigator and the medical monitor or designee
- Confirmed PR interval >260 ms (severe first-degree atrioventricular block)
- Confirmed second- or third-degree heart block

The investigator could have also, at their discretion, discontinued the participant from participating in the study at any time.

Participants requesting to discontinue study drug remained in the study and continued to be followed for protocol-specified follow-up procedures. Participants who discontinued study drug may have continued to be followed for resolution of a pregnancy or serious adverse event.

# Supplemental Methods: Infections and Infestations Preferred Terms Excluded From the Analysis

Preferred terms of special interest (PTSIs) associated with non-specific infections and infections that were non-serious and/or typically not related to immunosuppression (eg, high-level terms including dental and oral soft tissue infections, ear infections, eye and eyelid infections, upper respiratory tract infections, urinary tract infections, viral infections not elsewhere classified) were excluded from this analysis. The full list of excluded terms is as follows:

- Vector-borne transmission of infection
- Asymptomatic bacteriuria
- Bacterial vaginosis
- Conjunctivitis bacterial
- Folliculitis
- Laryngitis bacterial
- Pharyngitis bacterial
- Respiratory tract infection bacterial
- Sinusitis bacterial
- Skin bacterial infection
- Tonsillitis bacterial
- Lyme disease
- Campylobacter gastroenteritis
- Urinary tract infection enterococcal
- Escherichia infection
- Escherichia urinary tract infection
- Chancroid
- Helicobacter gastritis
- Helicobacter infection
- Gonorrhea
- Oropharyngeal gonococcal infection
- Gastroenteritis shigella
- Shigella infection
- Furuncle
- Staphylococcal pharyngitis
- Erysipelas
- Pharyngitis streptococcal
- Condyloma latum
- Latent syphilis
- Primary syphilis
- Secondary syphilis
- Syphilis
- Anal chlamydia infection
- Chlamydial infection
- Urethritis chlamydial
- Acrodermatitis
- Bed bug infestation
- Demodicidosis
- Balanitis candida
- Candida infection
- Skin candida
- Vulvovaginal candidiasis
- Fungal infection
- Fungal skin infection
- Genital infection fungal
- Onychomycosis
- Vulvovaginal mycotic infection
- Body tinea
- Dermatophytosis
- Tinea cruris
- Tinea infection
- Tinea pedis
- Helminthic infection
- Strongyloidiasis
- Appendicitis
- Diarrhea infectious
- Diverticulitis
- Dysentery
- Enteritis infectious
- Breast abscess
- Oral infection
- Periodontitis
- Pulpitis dental
- Tooth abscess
- Tooth infection
- Ear infection
- Otitis externa
- Otitis media
- Otitis media acute
- Otitis media chronic
- Conjunctivitis
- Eye infection
- Hordeolum
- Vaginal infection
- Vulval abscess
- Vulvovaginitis
- Abscess
- Abscess limb
- Device-related infection
- Postoperative wound infection
- Pyuria
- Wound infection
- Epididymitis
- Soft tissue infection
- Carbuncle
- Impetigo
- Pustule
- Pyoderma
- Rash pustular
- Skin infection
- Subcutaneous abscess
- Laryngitis
- Nasopharyngitis
- Peritonsillar abscess
- Pharyngitis
- Pharyngotonsillitis
- Rhinitis
- Sinusitis
- Tonsillitis
- Tracheitis
- Upper respiratory tract infection
- Cystitis
- Urethritis
- Urinary tract infection
- Vascular device infection
- Trichomoniasis
- Gastroenteritis norovirus
- Norovirus infection
- Herpangina
- Dengue fever
- Oral herpes
- Influenza
- Molluscum contagiosum
- Cervicitis human papilloma virus
- Papilloma viral infection
- Respiratory syncytial virus infection
- Rhinovirus infection
- Arthritis viral
- Conjunctivitis viral
- Gastroenteritis viral
- Respiratory tract infection viral
- Viral infection
- Viral pharyngitis
- Viral rhinitis
- Viral skin infection
- Viral tonsillitis

# Supplementary Figures and Tables

## Supplementary Figure


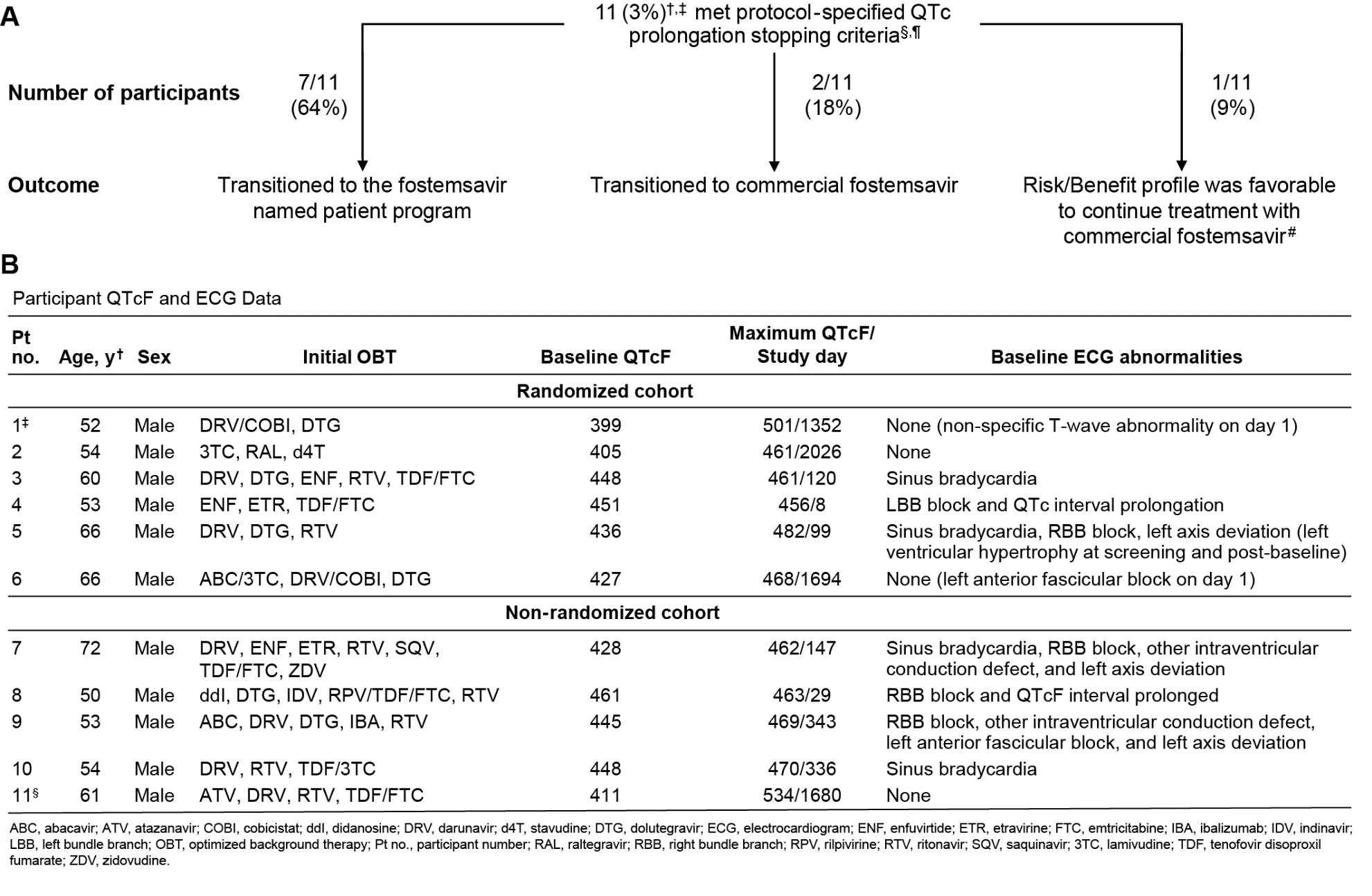


**Supplementary Figure 1.** **(A)** Outcomes in participants who met protocol-specified QTc prolongation stopping criteria. 10/11 participants had access to continued treatment with fostemsavir, and 9 were known to have continued fostemsavir. ^†^7 participants discontinued through the week 96 cutoff and an additional 4 discontinued through the week 240 cutoff. In 6 cases, 1 or more baseline ECG abnormalities were present, including left or right bundle branch block (n=5), sinus bradycardia (n=4), and QTc >450 ms (n=2). ^‡^In 4 cases, a non-serious adverse event of ECG QT prolongation was reported by the investigator. There was no protocol requirement for the investigator to report a QTc prolongation event as a clinical adverse event. ^§^Confirmed QTcF >470 ms in female participants and >450 ms in male participants, or confirmed QTcF increase of >60 ms over baseline. ^¶^No cases of symptomatic cardiovascular disease were reported. ^#^Transition not confirmed. **(B)** Participant QTcF data and baseline ECG abnormalities. ^†^Age at enrolment. ^‡^Participant with atrial fibrillation and treated with amiodarone. ^§^Right bundle branch block observed post-baseline at time of QTcF prolongation.

## Supplementary Tables

| **Supplemental Table 1.** Demographic and Baseline Disease Characteristics (Safety Population) | | | |
| --- | --- | --- | --- |
| **Characteristic** | **Randomized cohort (N=272)** | **Non-randomized cohort (N=99)** | **Total  (N=371)** |
| Age, n (%), years  <35  35-49  ≥50 | 61 (22)  100 (37)  111 (41) | 14 (14)  30 (30)  55 (56) | 75 (20)  130 (35)  166 (45) |
| Sex, n (%)  Male  Female | 201 (74)  71 (26) | 89 (90)  10 (10) | 290 (78)  81 (22) |
| Race, n (%)  White  Black or African American  American Indian or Alaska Native  Asian  Native Hawaiian or Other Pacific Islander  Other races | 185 (68)  60 (22)  7 (3)  2 (<1)  1 (<1)  17 (6) | 74 (75)  23 (23)  1 (1)  0  0  1 (1) | 259 (70)  83 (22)  8 (2)  2 (<1)  1 (<1)  18 (5) |
| HIV-1 RNA, median (IQR),  log_10_ copies/mL | 4.66 (3.87-5.09) | 4.31 (3.64-4.77) | 4.55 (3.85-5.02) |
| Baseline HIV-1 RNA, n (%), copies/mL  <1000  1000 to <10,000  10,000 to <100,000  ≥100,000 | 31 (11)  44 (16)  117 (43)  80 (29) | 9 (9)  24 (24)  51 (52)  15 (15) | 40 (11)  68 (18)  168 (45)  95 (26) |
| CD4+ T-cell count, median (IQR), cells/mm^3^ | 99.5 (15-207) | 41.0 (6-161) | 80.0 (11-202) |
| Baseline CD4+ T-cell count, n (%), cells/mm^3^  <20  20 to <50  50 to <100  100 to <200  ≥200 | 72 (26)  25 (9)  39 (14)  63 (23)  73 (27) | 40 (40)  14 (14)  14 (14)  11 (11)  20 (20) | 112 (30)  39 (11)  53 (14)  74 (20)  93 (25) |
| Number of years treated for HIV, n (%)  ≤10  11-15  16-20  >20  Unknown | 41 (15)  44 (16)  90 (33)  92 (34)  5 (2) | 5 (5)  11 (11)  22 (22)  58 (59)  3 (3) | 46 (12)  55 (15)  112 (30)  150 (40)  8 (2) |
| FAA ARVs in initial OBT, n (%)  0  1  2 | 15 (6)  142 (52)  115 (42) | 79 (80)  20 (20)^†^  0 | 94 (25)  162 (44)  115 (31) |
| ARV, antiretroviral; FAA, fully active and available; OBT, optimized background therapy.  ^†^4 participants had 1 fully active and available ARV at screening, and 16 received ibalizumab, which was still investigational at study start. | | | |

| **Supplemental Table 2.** Summary of Drug-Related Serious Adverse Events | | | |
| --- | --- | --- | --- |
| **System organ class and preferred term, n (%)** | **Randomized cohort (N=272)** | **Non-randomized cohort (N=99)** | **Total  (N=371)** |
| Any drug-related serious adverse event | 10 (4) | 3 (3) | 13 (4) |
| Renal and urinary disorders | 2 (<1) | 1 (1) | 3 (<1) |
| Nephrolithiasis | 1 (<1) | 1 (1) | 2 (<1) |
| Acute kidney injury | 1 (<1)^†^ | 0 | 1 (<1) |
| Cardiac disorders | 1 (<1) | 1 (1) | 2 (<1) |
| Myocarditis | 0 | 1 (1) | 1 (<1) |
| Supraventricular tachycardia | 1 (<1) | 0 | 1 (<1) |
| Immune system disorders | 2 (<1) | 0 | 2 (<1) |
| IRIS | 2 (<1)^‡, §^ | 0 | 2 (<1) |
| Metabolism and nutrition disorders | 1 (<1) | 1 (1) | 2 (<1) |
| Hyperglycemia | 1 (<1) | 0 | 1 (<1) |
| Hyperkalemia | 0 | 1 (1) | 1 (<1) |
| Nervous system disorders | 1 (<1) | 1 (1) | 2 (<1) |
| CNS immune reconstitution inflammatory response | 0 | 1 (1) | 1 (<1) |
| Loss of consciousness | 1 (<1)^¶^ | 0 | 1 (<1) |
| Hepatobiliary disorders | 1 (<1) | 0 | 1 (<1) |
| Hepatocellular cytolysis | 1 (<1)^§^ | 0 | 1 (<1) |
| Musculoskeletal and connective tissue disorders | 1 (<1) | 0 | 1 (<1) |
| Rhabdomyolysis | 1 (<1)^§^ | 0 | 1 (<1) |
| Pregnancy, puerperium, and perinatal conditions | 1 (<1) | 0 | 1 (<1) |
| Fetal growth restriction | 1 (<1)^‡^ | 0 | 1 (<1) |
| Psychiatric disorders | 1 (<1) | 0 | 1 (<1) |
| Disorientation | 1 (<1)^¶^ | 0 | 1 (<1) |
| Skin and subcutaneous tissue disorders | 1 (<1) | 0 | 1 (<1) |
| Rash | 1 (<1) | 0 | 1 (<1) |
| CNS, central nervous system; IRIS, immune reconstitution inflammatory syndrome.  ^†^Acute kidney injury (day 62 onset) and renal impairment (day 70 onset) occurred in the same individual. ^‡^1 of the IRIS cases (day 26 onset) and fetal growth restriction (day 559 onset) occurred in the same individual. ^§^3 participants discontinued from the study due to a drug-related serious adverse event (IRIS, hepatocellular cytolysis, and rhabdomyolysis). ^¶^Loss of consciousness and disorientation (day 147 onset for both) occurred in the same individual. | | | |

| **Supplemental Table 3.** All Deaths Through Week 240 | | | | |
| --- | --- | --- | --- | --- |
| **Cause of death** | | | **CD4+ T-cell count (cells/mm^3^)** | |
| **Category** | **Primary cause** | **Study day of death** | **Baseline** | **Last before death** |
| **Randomized cohort (n=15)** | | | | |
| AIDS-related (4/15 [27%]) | Pneumonia | 1821 | 92 | 171 |
|  | Pneumonia | 199 | 1 | 1 |
|  | Anal squamous cell carcinoma^†^ | 765 | 249 | 257 |
|  | Progressive multifocal leukoencephalopathy | 287 | 55 | 55 |
| Acute infections  (8/15 [53%]) | Pulmonary septic shock | 1266 | 5 | 0 |
|  | Meningoencephalitis viral | 101 | 11 | 8 |
|  | Septic shock^‡^ | 334 | 12 | 37 |
|  | Acute respiratory failure (community-acquired pneumonia)^‡^ | 11 | 14 | 14 |
|  | IRIS^§^ | 32 | 1 | 5 |
|  | Pseudomonal sepsis | 603 | 160 | 172 |
|  | Staphylococcal sepsis | 466 | 166 | 176 |
|  | Pneumonia^†^ | 228 | 1 | 1 |
| Non-AIDS malignancies (3/15 [20%]) | Rectal cancer^¶^ | NR | 98 | 160 |
|  | Squamous cell carcinoma^†^ | 535 | 27 | 30 |
|  | Cholangiocarcinoma | 875 | 349 | 494 |
| **Non-randomized cohort (n=20)** | | | | |
| AIDS-related (8/20 [40%]) | HIV wasting syndrome | 1112 | 1 | 4 |
|  | Progression of AIDS disease | 1772 | 80 | 2 |
|  | Acute kidney injury (advanced AIDS) | 350 | 1 | 4 |
|  | Disseminated cytomegaloviral infection | 661 | 1 | 3 |
|  | Cytomegalovirus colitis^†^ | 354 | 3 | 1 |
|  | Kaposi’s sarcoma | 160 | 2 | 10 |
|  | Encephalitis cytomegalovirus | 158 | 4 | 6 |
|  | Lymphoma | 33 | 42 | 24 |
| Acute infections (4/20 [20%]) | Clostridium difficile colitis | 511 | 1 | 3 |
|  | Sepsis | 504 | 7 | 37 |
|  | Sepsis | 580 | 35 | 18 |
|  | Septic rash (pulmonary/ cutaneous sepsis) | 392 | 42 | 234 |
| Non-AIDS malignancies (3/20 [15%]) | Hodgkin’s disease | 660 | 0 | 5 |
|  | Hodgkin's disease | 1094 | 28 | 148 |
|  | Tonsil cancer^†^ | 515 | 173 | 7 |
| Other causes (5/20 [25%]) | Dyspnea | 1089 | 4 | 1 |
|  | Hepatic failure (due to hepatitis B) | 537 | 1 | 2 |
|  | Hepatic failure | 142 | 1 | 2 |
|  | Cardiovascular disorder | 530 | 6 | 34 |
|  | Cerebrovascular accident (left middle cerebral artery stroke) | 879 | 22 | 189 |
| ^†^5 deaths occurred after the participant discontinued from the study. ^‡^Participants randomly assigned to placebo during the double-blind period. ^§^Death was considered treatment related (recurrent atypical mycobacterial infection due to immune reconstitution inflammatory syndrome). ^¶^Date of death was unknown; last CD4+ T-cell count was measured on study day 1387. | | | | |

| **Supplemental Table 4.** Characteristics of the 7 Participants Hospitalized With COVID-19 | | | | | | | |
| --- | --- | --- | --- | --- | --- | --- | --- |
| **Demographics and baseline characteristics** | | | | | | | |
| Age, years | 54 | 47 | 38 | 71 | 55 | 55 | 62 |
| Sex | Female | Male | Male | Male | Male | Female | Male |
| Race | Black | Other race | White | Black | Other race | White | White |
| Country | Brazil | Peru | Argentina | Belgium | Brazil | Brazil | Argentina |
| CD4+ T-cell count, cells/mm^3^ | 75 | 196 | 131 | 207 | 7 | 368 | 222 |
| HIV-1 RNA, copies/mL | 82,270 | 25,694 | 373,289 | 2395 | 112,343 | 54,925 | 346,054 |
| **Pre–COVID-19 clinical characteristics** | | | | | | | |
| CD4+ T-cell count, cells/mm^3^ | 823 | 293 | 876 | 310 | 563 | 1641 | 164 |
| HIV-1 RNA, copies/mL | <40 | <40 | <40 | <40 | 117 | <40 | 419,183 |
| **COVID-19 clinical characteristics** | | | | | | | |
| Positive test date | 15 Apr 2020 | 20 May 2020 | 07 Jul 2020 | 26 Oct 2020 | 12 Feb 2021 | 09 Mar 2021 | 31 Mar 2021 |
| Event duration, days | 16 | 19 | 19 | 15 | 17 | 43 | 22 |
| Severity | Grade 3 | Grade 3 | Grade 2 | Grade 3 | Grade 3 | Grade 2 | Grade 3 |
| Outcome | Recovered | Recovered | Recovered | Recovered | Recovered | Recovered | Recovered |
| Relevant medical history or known exposure risks | Diabetes, SAH, no known exposure | SAH, obesity, recent community exposure | Smoker, no known exposure | Chronic renal failure/dialysis, recent visit to  healthcare facility | SAH, recent community exposure | Asthma, recent community exposure + visit to healthcare facility | No known exposure |
| Reported COVID-19 treatment | Ceftriaxone IV, azithromycin, oseltamivir, cefuroxime, enoxaparin, supplemental oxygen via nasal catheter | Orphenadrine, acetaminophen, enoxaparin, albuterol, ipratropium | Enoxaparin, omeprazole | Dexamethasone, enoxaparin, aspirin, tramadol, movicol, furosemide, bilastine, darbepoetin alfa, valproic acid, potassium, sodium bicarbonate, calcium carbonate, supplemental oxygen via nasal canula | Ceftriaxone, ciprofloxacin, dexamethasone, enoxaparin, loperamide, oxygen support | Dexamethasone,  oxygen support | Blood transfusion, ampicillin/ sulbactam, budesonide inhaler, ipratropium inhalation |
| IV, intravenous; SAH, systemic arterial hypertension. | | | | | | | |

| **Supplemental Table 5.** On-Treatment Events of Immune Reconstitution Inflammatory Syndrome (Safety Population) | | | | | |
| --- | --- | --- | --- | --- | --- |
| **Onset day^†^** | **Maximum grade** | **Serious** | **Related to study drug** | **CD4+ T-cell count, cells/mm^3‡^** | **Comments** |
| 42 | 2 | No | Yes | Baseline, 3 Day 36, 397 | CNS lesion; progressive multifocal  leukoencephalopathy |
| 87 | 2 | No | Yes | Baseline, 3 Day 87, 43 | History of HCV (RNA positive at study entry) and cryptococcal meningitis |
| 109 | 3 | No | Yes | Baseline, 55 Day 93, 165 | Progressive multifocal  leukoencephalopathy, pneumococcal  pneumonia |
| 4 | 2 | No | Yes | Baseline, 5  Day 8, 9 | Immune reconstitution folliculitis |
| 32 | 4 | Yes | Yes | Baseline, 1 Day 8, 5 | Atypical mycobacterial infection, fatal |
| 26 | 3 | Yes | Yes | Baseline, 281 Day 8, 364 | Cerebral toxoplasmosis |
| 44 | 2 | No | No | Baseline, 13 Day 30, 90 | History of CMV colitis and retinitis, and cerebral toxoplasmosis |
| 3 | 3 | Yes | Yes | Baseline, 73 Day 31, 70 | IRIS-related CNS lesions, not otherwise specified; past history of JC virus infection |
| CMV, cytomegalovirus; CNS, central nervous system; HCV, hepatitis C virus; IRIS, immune reconstitution inflammatory syndrome; JC, John Cunningham.  ^†^Relative to first dose of study drug. ^‡^CD4+ T-cell count at baseline and closest to time of reported IRIS. | | | | | |
